# Assessment of the Sinus Septa Using CBCT: A Cross-Sectional Study in the Asir-Region Population, Abha Residents

**DOI:** 10.3390/jcm14248784

**Published:** 2025-12-11

**Authors:** Hassan Ahmed Assiri, Atheer Almuaddi, Reema Malwi, Norah Alwadai, Ali Azhar Dawasaz, Abdullah Alqarni, Saeed Alassiri

**Affiliations:** 1 Department of Diagnostic Sciences and Oral Biology and Periodontology, College of Dentistry King Khalid University, Abha 61421, Saudi Arabia; 2 Internship Program, College of Dentistry, King Khalid University, Abha 61421, Saudi Arabia; 3 Department of Dental Research, Dr. D. Y. Patil Dental College & Hospital, Dr. D. Y. Patil Vidyapeeth (Deemed to be University), Pune 411018, India

**Keywords:** maxillary sinus, sinus septa, maxilla, cone-beam computed tomography

## Abstract

**Background**: Anatomical variations of the maxillary sinus, including the septa, can affect surgical outcomes. This study aimed to present the characteristics of maxillary sinus septa in an Asir-region cohort using cone-beam computed tomography (CBCT). **Methods**: Archival adult CBCT scans of patients at the King Khalid University College of Dentistry were reviewed in this retrospective cross-sectional study. Septa were measured in axial, coronal, and sagittal planes and classified as vertical, oblique, or horizontal. Correlations between the characteristics of the septa and both age and sex were analyzed. **Results**: Of the 400 CBCTs randomly selected between May–August 2025, 350 were suitable for analysis; among them, only 26 patients (53.8% male; age, <30 years) presented with sinus septa. The septa were unilateral in 16 (61.5%) and bilateral in 10 (38.5%) patients, without any significant differences based on sex (χ^2^ = 0.248; *p* = 0.619) or age (χ^2^ = 5.491; *p* = 0.139). Oblique and horizontal orientations were most common on the right (*n* = 10) and left (*n* = 11) sides, respectively; no associations were observed with sex (*p* > 0.05). The mean mediolateral length and septal height ranged from 7.8 to 10.3 mm and ~8.2 to 8.5 mm, with no sex- or age-related differences. Septal thickness did not vary by age (*p* > 0.05). Pathologic mucosal findings were infrequent and showed no association with septal location or side. **Conclusions**: These findings provide region-specific data on maxillary sinus septa in the Asir-region cohort, contributing to the understanding of anatomical variations before planning surgical interventions.

## 1. Introduction

Cone-beam computed tomography (CBCT) is crucial for the detailed visualization of relevant anatomical structures, facilitating accurate diagnosis and treatment planning due to its high spatial resolution and minimal radiation exposure relative to conventional radiographs [1,2,3]. The maxillary sinus, an anatomical cavity within the facial skeleton, represents the largest among the paranasal sinuses, including the frontal, ethmoidal, and sphenoidal sinuses. It plays an essential role in reducing skull weight, aids in warming and humidifying inhaled air, and influences voice resonance [4]. However, its anatomical complexity and variability, including traits such as septa, sinus hypoplasia/hyperplasia, and accessory ostia, may affect surgical outcomes and necessitate meticulous preoperative assessment [5]. Evaluation of the maxillary sinus and its septa is crucial for diagnostic accuracy in implant dentistry, sinus lift procedures, and oral and maxillofacial surgery. The incidence, orientation, and position of maxillary sinus septa are variable and may complicate treatments like sinus lift and implant insertion [6,7]. Studies utilizing CBCT have demonstrated significant heterogeneity in septal architecture at the population level. Pommer et al. [8] documented a prevalence range of 20–58%, exhibiting considerable variations within cohorts, whereas Al Zahrani et al. [9] reported an approximate prevalence of 36% in a Saudi population, highlighting the influence of regional factors. In an East Asian cohort, Shen et al. [10] reported a prevalence of approximately 50%, underscoring the significance of three-dimensional CBCT assessment for pre-surgical planning. Recent systematic reviews and large CBCT studies confirm the varied incidence and morphology, including geographic patterns and limited demographic correlations, and expand upon these foundational studies [11,12,13]. CBCT facilitates direct visualization of septal height and orientation, as well as relationships with adjacent bony structures, which can significantly influence clinical decisions and mitigate complications. Additionally, it enhances the detection of subtle sinus findings, such as mucosal thickening and antral pseudocysts, which are pertinent to implant planning [7,14]. Recent evidence from 2020 to 2025 further substantiates these diagnostic benefits [15]. The clinical significance of septa is closely linked to the planning of sinus augmentation. In the presence of septa, Schneiderian membrane perforation rates can increase roughly 2–3 folds as studies reported. Furthermore, the location of lateral windows and the feasibility of a single window approach can be influenced by the septal numbers, height, and orientation. In endoscopic surgery, the identification of the septa by three-dimensional imaging can reduce the risk of iatrogenic injury [16,17]. Current guidelines advocate a CBCT-first approach, emphasize indications for lateral-window techniques, and identify anatomical risk modifiers (e.g., tall/oblique septa) that guide operative planning based on both classical and recent evidence [16,18,19,20]. To our knowledge, no previous study has explored the occurrence and morphometric characteristics of sinus septa among residents of Abha in the Asir region of Saudi Arabia that can influence the surgical intervention. Craniofacial morphology is known to differ across populations, highlighting the need for localized anatomical data. Therefore, this study employed CBCT to analyze the anatomical features and morphometric variations of maxillary sinus septa in the Abha population and provide core evidence that guide different surgical procedures.

## 2. Materials and Methods

### 2.1. Study Design and Setting

This was a single-center, retrospective cross-sectional study of archival CBCT scans of patients who attended the dental hospital at King Khalid University (KKU-COD) from May to August 2025, representing a cohort of residents from Abha city in the Asir region of Saudi Arabia. The research was conducted in accordance with the Declaration of Helsinki and approved by the Internal Review Board of King Khalid University; protocol number (KKU-28-2025-14, approval date 19 May 2025) [21]. Archived scans were randomly selected from patients who had previously received CBCT imaging for various therapeutic purposes (e.g., implant planning, orthognathic surgery, pathology delineation, and impacted teeth evaluation) using a computer-generated random-number sequence to ensure unbiased selection. The primary outcome was the presence of septa with a minimum height of ≥2 mm while the other outcomes included the septum laterality, height, thickness, and orientations. In addition, the association of these outcomes with sex and genders were reported.

### 2.2. Eligibility Criteria

We included artifact-free CBCT scans of patients aged 18 years and older, with no maxillary sinus disease that could impair visualization or morphometric assessment of septa. Scans with an inadequate field of view or subpar image quality that failed to visualize the entire sinus were excluded.

### 2.3. Sample Collection

A total of 400 CBCT scans were randomly selected; after applying the eligibility criteria, 350 patients qualified for inclusion. The age range of the included cohort is between (18–70 years old). The sample size was calculated a priori based on an expected prevalence of 36% from Al-Zahrani et al. [9], with a 5% margin of error, a 95% confidence level (α = 0.05), and 80% power, resulting in a target sample size of 350 patients.

### 2.4. Radiographic Measurements

**Figure 1:**
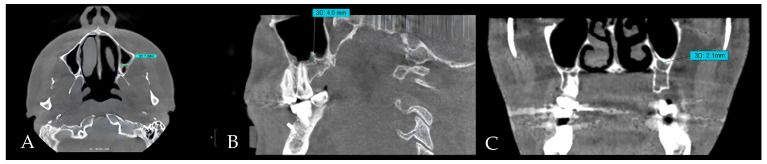
CBCT images of the maxillary sinus septa. (**A**) Mediolateral diameter of the maxillary sinus septa depicted on the axial plane. (**B**) The height of the observed septa measured on the sagittal plane. (**C**) The thickness of the observed septa measured at its thickest part.

All scans were obtained using a KaVo OP 3D Pro CBCT machine (Planmeca, Biberach, Germany). Measurements were conducted using OnDemand3D^®^ software version 1.0 (Build 1.0.10.7462) (x64 Edition). The specified field of view was 8 × 15 cm, with acquisition settings (90 kVp, 6.3 mA, and 4.5 s), and a voxel size of 0.2 mm. For calibration, 15% of the images were initially evaluated to verify measurement consistency. To minimize the risk of bias, the pilot scans were excluded from the final analyzed images. Septal length, orientation, and thickness were measured by three authors: one oral and maxillofacial radiologist and two dental interns (Figure 1A–C). All identified images were subsequently re-evaluated. Inter-rater agreement was assessed by conducting repeated measures after one month and calculating Cohen’s κ coefficient. The sinus septum was defined as a bony ridge projection originating from the wall or floor of the sinus. The minimum detectable height was ≥2 mm [22]. The morphology of the septum was characterized based on the orientation (vertical, oblique, or horizontal), mediolateral length (on the axial plane; Figure 1A), height (on the sagittal plane; Figure 1B), and thickness (Figure 1C). Three calibrated examiners conducted independent measurements; the final values utilized for analysis were established by consensus rather than average, subsequent to a review of differences under the radiologist’s oversight.

### 2.5. Statistical Analysis

Data were entered into Microsoft Excel for Microsoft 365 (Microsoft Corp., Redmond, WA, USA) version 2509 and analyzed using Stata 19 (StataCorp LLC, College Station, TX, USA). The Mann–Whitney U test was used to compare septal numbers between males and females, for the right and left sides, and the Kruskal–Wallis test was used for age. Independent samples *t*-tests and analysis of variance (ANOVA) F-tests were performed to compare septal thickness between males and females, as well as among the various age groups, respectively. Providing the nature of the study and the relatively small number of septa identified, *p*-values were reported with cautions with no adjustments for multiple comparisons

### 2.6. Agreement and Reliability

After a collaborative training session with 15 pilot scans, three calibrated examiners (one oral & maxillofacial radiologist and two dental interns) independently performed all measurements. Approximately 15% of the scans were reassessed after one-month to quantify the intra-observer and interobserver agreement. A two-way random-effects ICC (absolute agreement) was computed for continuous variables and a weighted κ for categorical variables (e.g., orientation). The interobserver reliability exhibited an ICC of 0.77 (good) and a κ of 0.58 (moderate). Any discrepancies among examiners were resolved through joint review and consensus discussion under the supervision of the oral and maxillofacial radiologist.

## 3. Results

Demographics: A total of 350 CBCTs screened for the presence of septa. Among them, only 26 participants presented with at least on maxillary sinus septum. Fourteen of them (53.8%) males and twelve (46.2%) females, resulting in an approximate ratio of 1:1. The majority (61.5%) were under 30 years of age; 15.4% were aged 41–50 years (Table 1). The overall prevalence of the septa was 7.43%

Laterality and distribution: The distribution of unilateral and bilateral septa exhibited no significant difference by sex (χ^2^ = 0.248; *p* = 0.619). Unilateral septa occurred more frequently in younger participants, while bilateral septa were predominantly observed in older groups, albeit not to a statistically significant extent (χ^2^ = 5.491; *p* = 0.139). Anterior and posterior bilateral septa predominated on the left side, whereas unilateral septa were predominantly midline, exhibiting a borderline tendency (χ^2^ = 5.439; *p* = 0.066). No significant differences were noted on the right side (χ^2^ = 0.830; *p* = 0.660) (Table 2).

Number of septa by age and sex: Nonparametric tests were used to assess the influence of sex and age on maxillary sinus septa measurements. The Mann–Whitney U test revealed no significant differences between males and females for either the right (U = 78.5, *p* = 0.756) or left (U = 41.0, *p* = 0.726) sides. Similarly, the Kruskal–Wallis test revealed no significant differences among age groups for the right (H = 5.335, *p* = 0.149) or left (H = 1.280, *p* = 0.734) sides. These results suggest that septa characteristics were not significantly affected by sex or age in this cohort.

Dimensions: The mediolateral length on the right side was greater in males, although the differences were not statistically significant (t = 1.516; *p* = 0.150 for the right side; *p* = 0.954 for the left side). No substantial age effects were observed (right F = 0.773; *p* = 0.480; left F = 0.511; *p* = 0.681). No significant differences in the vertical height were observed based on sex (right *p* = 0.931; left *p* = 0.831) or age (right *p* = 0.442; left *p* = 0.969) (Table 3).

Orientation: The right-side orientation exhibited no notable changes based on sex (χ^2^ = 2.871; *p* = 0.238) or age (χ^2^ = 5.667; *p* = 0.225). The left side orientation showed no significant variations based on sex (χ^2^ = 2.392; *p* = 0.302) or age (χ^2^ = 6.932; *p* = 0.327). Younger participants exhibited a predominance of oblique orientation on the right, while horizontal orientation was most prevalent on the left (Table 4). Overall, these dimensions beside the orientations of the septa in this cohort require modification of lateral window technique or endoscopic surgery approach.

Pathology distribution: Pathology was marginally more prevalent in anterior portions; however, none of the comparisons achieved statistical significance (right side by location: χ^2^ = 0.744; *p* = 0.689; left vs. right: χ^2^ = 0.635; *p* = 0.728; left side by location: χ^2^ = 0.684; *p* = 0.710).

Thickness: ANOVA revealed no significant variations in septal thickness across age groups for the left (F = 0.379; *p* = 0.691) or right (F = 0.754; *p* = 0.537) sides (Table 5).

## 4. Discussion

This cross-sectional CBCT analysis of patients from the Asir region demonstrated the presence of maxillary sinus septa across sexes, age groups, and sinus sites, with no statistically significant associations identified. Unilateral septa were more prevalent in younger individuals, while bilateral septa were more commonly observed in older participants; nevertheless, neither trend exhibited statistical significance. This overarching pattern—characterized by the presence of septa with weak or nonexistent demographic correlations—corresponds to previous CBCT-based syntheses and observational studies, indicating considerable population heterogeneity but limited and inconsistent relationships with sex or age [5,7,8,10]. Regional and East Asian cohorts likewise demonstrated variability without strong demographic predictors [7,9]. Our findings underscore the significance of CBCT for preoperative sinus evaluation in implant dentistry and sinus augmentation. In contrast to two-dimensional imaging, CBCT provides authentic three-dimensional visualization and enhanced spatial resolution, facilitating the detection of septa (including minor or secondary ridges) and allowing precise evaluation of septal height, thickness, and orientation [1,3,6,14]. These advantages directly influence surgical planning, as undetected septa may complicate lateral-window design and elevate the risk of Schneiderian membrane perforation [2]. The observed prevalence of maxillary sinus septa in our study (7.43%) is notably lower than the 20–50% reported in previous studies. This discrepancy may be attributable to several methodological factors. First, the voxel size of CBCT imaging can influence septa detection, as smaller or thinner septa may not be visualized with larger voxel dimensions. Second, our strict inclusion criteria—excluding scans with artifacts, prior surgery, or incomplete sinus visualization—may have resulted in a more conservative estimate by omitting cases in which septa might have been present but unmeasurable. Finally, population-specific anatomical variations could also contribute to the lower prevalence, emphasizing the need for regionally focused studies A lower septal prevalence in this cohort suggests a sinus anatomy with fewer internal bony interruptions, which may allow smoother membrane elevation during sinus-lift procedures and reduce the likelihood of perforation.

Regarding orientation, our sample exhibited a prevalence of oblique septa on the right among younger participants and more horizontal septa on the left, albeit without statistical significance. The literature reveals that oblique septa are generally predominant, with variations in side-specific or age-related patterns among different cohorts [5,7]. For example, the systematic review by Pommer et al. found that the transverse orientation was present in approximately 87.6% of septa, with sagittal and horizontal orientations being significantly less common [8]. Collectively, these findings emphasize the importance of individualized CBCT evaluation of each sinus prior to surgical procedures, as septal orientation may vary between the right and left sides, potentially impacting sinus lift or other maxillary interventions. Considering this diversity, personalized CBCT mapping of septal orientation and location is crucial for determining the safest surgical approach—modifying the configuration or number of lateral windows or revising the antrostomy position—to minimize membrane injury [2,6]. In the present study, the mediolateral length (7.7–10.2 mm) and height (8.2–8.5 mm) were somewhat higher than those reported in previous studies (4.6–5.4 mm for mediolateral length and 1.6–13.1 mm for septal height), indicating substantial interindividual variability and minimal sex effects on septal size [7,8]. These findings highlight the importance of preoperative CBCT evaluation for all patients, as taller septa, irrespective of age or sex, can increase the risk of membrane perforation during sinus lift procedures. Clinically, the focus should remain on a patient-specific morphology. Taller, sharper, or unfavorably positioned septa may require adjusted window placement or a staged approach to reduce the risk of perforation [3,6]. Our data indicate that clinically significant maxillary sinus septa (≥2 mm height) are found in approximately 1 in 13 patients undergoing CBCT in this context. We did not gather intraoperative outcomes; however, previous clinical studies and systematic reviews consistently identify septa as a significant risk factor for Schneiderian membrane perforation during lateral window sinus floor elevation, frequently elevating perforation rates by two- to three-fold relative to sinuses devoid of septa. The morphometric profile of this group shows that many of these septa are tall enough to interfere with standard lateral window designs and require changes to the osteotomy (for example, double windows, narrowed or more posterior windows) or, in some cases, a different approach. In otolaryngology practice, preoperative computed tomography (CT) or CBCT is essential for functional endoscopic sinus surgery, as the identification of complete or high septa directs the placement of maxillary antrostomies and ensures complete access to posterior sinus compartments [16,17].

A marginally higher incidence of anterior sinus disease was noted; however, this difference did not reach statistical significance. This finding aligns with CBCT studies indicating that incidental mucosal changes (e.g., mucosal thickening and antral pseudocysts) are common among implant candidates; septa should therefore be regarded as anatomical variants rather than pathological indicators [5,14]. Thus, septal characterization must be combined with a comprehensive CBCT assessment of the ostiomeatal complex, sinus floor, and surrounding dentoalveolar structures to contextualize soft tissue observations [1,2,14]. Compared to earlier CBCT studies conducted in Saudi Arabia and neighboring regions [9,11,12], our findings provide additional region-specific information on the maxillary sinus septa. While the previous studies generally reported higher septal prevalence and a predominance of vertically oriented septa, the present study demonstrated a comparatively lower prevalence in the Asir-region cohort and a different distribution of orientations, where oblique septa were more common on the right and horizontal septa predominated on the left. Furthermore, although earlier studies noted possible trends related to age or sex, our analysis found no statistically significant demographic associations, indicating that septal characteristics in this population may be largely independent of these factors. Our study also provides updated quantitative measurements of septal length, height, and thickness using a standardized multiplanar CBCT protocol, offering contemporary reference values for this region. The morphometric values reported here offer useful reference points for anticipating membrane tension and selecting an appropriate surgical approach. As no age- or sex-related associations were identified, these findings underscore the importance of case-specific CBCT assessment in optimizing planning and improving safety in sinus augmentation procedures. Taken together, these distinctions expand the existing regional evidence base and provide clinicians with refined, locally relevant morphological data to support safer and more individualized sinus-related surgical planning.

This study has several limitations, including the single-center design, moderate sample size, and lack of stratification by dental status, a variable known to influence sinus remodeling and septation patterns [10]. The small number of septa identified (*n* = 26) limits the statistical power of the study. Additionally, the lack of data on dentition status could influence septal formation. Future research should incorporate multicenter samples, dentition-related characteristics, and prospective intraoperative outcomes (e.g., membrane perforation and augmentation success) to elucidate the relationship between distinct septal morphologies and surgical risk. Although previous studies have examined maxillary sinus septa in regional populations, our CBCT analysis of patients from the Asir region provides region-specific data. We observed a relatively low prevalence of septa (7.43%) across sexes, age groups, and sinus sites, with no statistically significant associations. These findings extend existing knowledge by highlighting potential influences of imaging parameters, inclusion criteria, and population-specific anatomical variation, thereby improving the understanding of sinus septa morphology in this population. Routine CBCT-based pre-surgical planning—identifying septal position and orientation, planning lateral windows accordingly, and anticipating technique modifications for tall or obliquely oriented septa—remains the most effective strategy to minimize complications and enhance outcomes in sinus augmentation and implant therapy [2,6,14].

## 5. Conclusions

In this Asir-region cohort, maxillary sinus septa were relatively infrequent (7.43%) and showed no association with age or sex; oblique and horizontal orientations were the most common patterns observed. When present, sinus septa were typically 8–10 mm long and 8 mm height with a predominance orientation of horizontal and oblique orientations. These findings highlight the anatomical variability of the sinus and underscore the importance of CBCT evaluation. Careful assessment of septal morphology and dimensions can aid in selecting an appropriate surgical approach, reducing the risk of membrane perforation, and improving the predictability of sinus-lift and implant procedures.

## Figures and Tables

**Table 1:** Demographic characteristics of the participants.

Characteristics	Frequency	Percentage
	(*n*)	(*n* %)
Gender Male	14	53.8
Female	12	46.2
Total	26	100
>30 years	16	61.5
30–40 years	3	11.5
Age 41–50 years	4	15.4
More than 50 years	3	11.5
Total	26	100

**Table 2:** Associations between unilateral/bilateral septa and other variables.

Study Variables	Unilateral	Bilateral	Total	χ^2^	*p*-Value
Sex					
Male	8	6	14	0.248	0.619
Female	8	4	12		
Age groups					
Age < 30	11	5	16	5.491	0.139
Age 30–40	3	0	3		
Age 41–50	1	3	4		
Age > 50	1	2	3		
Location of septa					
Left–Anterior	1	5	6	5.439	0.066
Left–Middle	5	1	6		
Left–Posterior	3	4	7		
Right–Anterior	5	5	10	0.830	0.660
Right–Middle	1	3	4		
Right–Posterior	1	2	3		

**Table 3:** Mediolateral length and vertical height of the septa in relation to age and sex.

Measurement	Group	Right Mean ± SD (mm)	Left Mean ± SD (mm)	Mean Diff	t	*p*-Value (Sex)	ANOVA (Age)	*p*-Value (Age)
Mediolateral length (axial plane)	Male	10.28 ± 3.11	7.99 ± 2.22	2.463	1.516	0.150	0.773	0.480
Mediolateral length (axial plane)	Female	7.82 ± 2.88	8.08 ± 4.16	–0.094	–0.058	0.954	0.511	0.681
Height (sagittal plane)	Male	8.23 ± 2.19	8.54 ± 3.02	–1.667	–0.087	0.931	0.866	0.442
Height (sagittal plane)	Female	8.40 ± 5.90	8.21 ± 3.29	0.328	0.217	0.831	0.081	0.969

**Table 4:** Orientation of maxillary sinus septa in relation to age and sex.

Side	Group	Vertical	Oblique	Horizontal	χ^2^	*p*-Value
Right side	Male	0	8	4	2.871	0.238
	Female	1	2	2		
	<30 years	1	8	2	5.667	0.225
	41–50 years	0	2	2		
	>50 years	0	0	2		
Left side	Male	2	3	3	2.392	0.302
	Female	1	2	8		
	<30 years	3	2	5	6.932	0.327
	30–40 years	0	1	2		
	41–50 years	0	2	1		
	>50 years	0	0	3		

**Table 5:** Septal thickness on both sides by age groups.

Septal Thickness		Sum of Squares	df	Mean Square	F	*p*-Value
Left side septa thickness	Between Groups	1.026	2	0.513	0.379	0.691
	Within Groups	18.929	14	1.352		
	Total	19.955	16			
Right side septa thickness	Between Groups	2.115	3	0.705	0.754	0.537
	Within Groups	14.034	15	0.936		
	Total	16.149	18			

## Data Availability

The original contributions presented in this study are included in the article. For further inquiries, please contact the corresponding authors.
